# Supplementary material for: Constant light and high fat diet alter daily patterns of activity, feed intake and fecal corticosterone levels in pregnant and lactating female ICR mice
Source: PLoS One. 2024 Nov 20;19(11):e0312419. doi: 10.1371/journal.pone.0312419 (PMC11578523; doi:10.1371/journal.pone.0312419)
Supplement: Attachment — Submitted filename: Response_to_Reviewers_08_30_2024.docx [file pone.0312419.s003.docx]

**Response to Manuscript Reviewers: “Constant light and high fat diet alter daily patterns of activity, feed intake and fecal corticosterone levels in pregnant and lactating female ICR mice”**

We would like to thank you for taking the time to review our manuscript, your comments served to be insightful and addressing them increased its quality.

The authors confirmed that the submission contains all raw data required to replicate the results of their study – S2 file.

**Reviewer #1:**

1) While there were no changes observed in cortisol, did you explore any of the other stress hormones/pathways? (ie, epinephrine, norepinephrine?), or photoperiod hormones (melatonin, prolactin?).

**Response:** Fecal corticosterone was measured as a biomarker of stress due to its well-established role in reflecting the hypothalamic-pituitary-adrenal (HPA) axis activity in rodents. We previously reported findings regarding serum prolactin (Teeple et al., 2023, doi:10.1242/BIO.060088/333487), which was significantly elevated in the LL treatment (see lines 65-69 in the Introduction). Melatonin was not measured in our study as a marker of photoperiod response, as ICR mice are homozygous for B6J *Hiomt* mutant alleles, rendering them unable to produce melatonin and thus melatonin deficient (Kasahara, T., et al 2010, doi.org/10.1073/pnas.0914399107).

2) There is no discussion about the phentoypes that can be observed during lactation where there is no actually adiposity ("lean" so to speak) in animals during lactation due to the energy associated with lactation, while sometimes there is. Where there any differences in adiposity between diets, diets*light exposure, or light exposure? How might this be related to outcomes of your study if at all?

**Response:** We have revised the manuscript (Lines 315-323) to better discuss these outcomes. In the present study, we focused on the effects of a high-fat diet and continuous light exposure on daily eating patterns, maternal activity, and corticosterone output. We previously addressed adiposity-related aspects in our prior studies (Teeple et al., 2023, doi:10.1242/BIO.060088/333487; Teeple et al., 2023, doi:10.1371/JOURNAL.PONE.0279209), finding that HF diet mice had higher BMI by lactation day 12, but adiposity differences diminished as they mobilized energy stores during lactation. Our findings align with the concept that HF dams, despite having higher BMI and percent body fat prior to lactation, mobilized their extra energy stores during lactation, leading to diminished differences in adiposity by the study's end. In contrast, CON mice likely had to rely on increased food consumption to meet the energy demands of lactation, which aligns with the observation that pre-pregnancy obesity in human models does not typically result in significant changes in BMI during lactation (Marshall et al., 2022 doi: 10.1016/j.ajog.2021.12.035; Winkvist and Rasmussen, 1999 doi: 10.1023/A:1018706131168).

3) Did you assess any maternal type behaviors? like pup retrieval and return to the nest? Were these affected by the treatments?

**Response:** We agree that assessment of maternal behaviors like pup retrieval and nest building would have strengthened the study, however, we decided to leave nesting material in the cage and limited behavior assessment to activity patterns in a relative non-invasive manner. We discuss this as a limitation of our study (lines 402-410, 420-434).
